# Experiences With a Novel Micro-Choice-Based Concentrated Group Intervention for People With Type 2 Diabetes: A Qualitative Study

**DOI:** 10.1177/26350106241304422

**Published:** 2025-02-03

**Authors:** Bente Elisabeth Bendixen, Ane Wilhelmsen-Langeland, Kirsten Lomborg, Eirin Måkestad, Trine L. Skogheim, Anne Schønberg, Marjolein M. Iversen, Gerd Kvale, Eirik Søfteland, Anne Haugstvedt

**Affiliations:** Department of Health and Caring Sciences, Western Norway University of Applied Sciences, Bergen, Norway; Department of Health and Caring Sciences, Western Norway University of Applied Sciences, Bergen, Norway; Helse i Hardanger, Øystese, Norway; Department of Psychiatry, Haukeland University Hospital, Bergen, Norway; Department of Health and Caring Sciences, Western Norway University of Applied Sciences, Bergen, Norway; Copenhagen University Hospital - Steno Diabetes Center Copenhagen, Copenhagen, Denmark; Department of Clinical Medicine, University of Copenhagen, Copenhagen, Denmark; Helse i Hardanger, Øystese, Norway; Helse i Hardanger, Øystese, Norway; Helse i Hardanger, Øystese, Norway; Department of Medicine, Haukeland University Hospital, Bergen, Norway; Department of Health and Caring Sciences, Western Norway University of Applied Sciences, Bergen, Norway; Helse i Hardanger, Øystese, Norway; Department of Psychiatry, Haukeland University Hospital, Bergen, Norway; Department of Clinical Psychology, Faculty of Psychology, University of Bergen, Bergen, Norway; Helse i Hardanger, Øystese, Norway; Department of Medicine, Haukeland University Hospital, Bergen, Norway; Department of Health and Caring Sciences, Western Norway University of Applied Sciences, Bergen, Norway; Helse i Hardanger, Øystese, Norway

## Abstract

**Purpose:**

The purpose of this study was to explore experiences with an interdisciplinary micro-choice-based concentrated group intervention for people with type 2 diabetes.

**Methods:**

A qualitative study with individual semistructured interviews were conducted with 14 adults (8 women, ages 45-74 years) with type 2 diabetes. Purposive sampling was used, and participants from 3 different intervention groups in the micro-choice-based concentrated group intervention were recruited. Thematic analysis was used for the data analysis.

**Results:**

Three main themes were identified: (1) group intervention tailored to individual needs through friendly and skilled professionals, (2) valuable social interactions and an experience of fellowship, and (3) commitment to change through goal setting and conscious micro-choices. The participants described a readiness for change that was met by important knowledge from skilled professionals in the concentrated intervention. They reported that new knowledge, particularly about micro-choices and the focus on how insulin works in the body, led to change in their awareness and self-management. The concentrated group intervention was a preferred setting that contained a sense of community without compromising on meeting individual needs. Participants described internalized changes after the intervention and a willingness to adhere to changes necessary for their self-management.

**Conclusion:**

Study findings showed that a micro-choice-based concentrated group intervention for people with type 2 diabetes can be a valuable approach contributing to improved patient activation and diabetes self-management. The findings underpin the importance of increased diabetes knowledge and support from an interprofessional team to bring about significant changes in everyday life.

Type 2 diabetes (T2DM) represents a rising health challenge globally both for individuals living with the disease and for health care systems.^1^ In Norway, only 3 in 5 persons with T2DM achieved the national treatment targets for glycemia (A1C < 53 mmol/mol).^2^ Diabetes self-management is a cornerstone to achieve satisfactory treatment outcomes, and people with T2DM need to make numerous choices and actions related to lifestyle every day, frequently including medical treatment decisions.^3,4^ For many, the self-management is perceived as complicated and demanding. Therefore, the diabetes self-management education (DSME) delivered by health care services is crucial. The DSME should be individually adapted in the same way as the medical and psychosocial follow-up and support should be. A person-centered follow-up is shown to improve health outcomes.^3^

In addition to the follow-up in health care services, people also seek information outside the health services, and they need to strain out which information is useful and which is not. The ability a person has to understand and use the health information they get from various sources and subsequently make informed decisions about their own health is defined as health literacy.^5^ Studies have shown that many people with T2DM have insufficient health literacy that hinders them from managing their condition satisfactorily.^6,7^ In addition to insufficient health literacy, the emotional burden and distress associated with T2DM have also been shown to be barriers for satisfactory self-management.^8^ Conversly, increased engagement and activation can reduce distress and improve self-management, which in turn can lead to improved treatment outcomes.^9-13^ A systematic review and meta-analysis has shown that group-based self-management education for individuals with T2DM is more effective than standardized care for improvements of lifestyle, psychosocial outcomes, and clinical outcomes.^14^ Several other group-based interventions have also been tested among people with T2DM, but they are often comprehensive and time-consuming both for the participants and the health care system.^15^ In addition, collaboration between health care professionals for people with T2DM ensures different perspectives and supports improved self-care and patient-satisfaction,^16^ advocating for an interdisciplinary approach in DSME. Thus, a cost-effective intervention that improves self-management and patient activation, reduces the psychosocial burden and diabetes distress, and subsequently improves treatment outcomes is highly warranted. Developing complex interventions should include an evaluation of context and participants’ perception to understand its interaction with the complex setting,^17^ hence the qualitative approach of this article, in which participants’ experiences with a novel interdisciplinary micro-choice-based concentrated group intervention for people with T2DM are presented.

## Methods

### Design

A qualitative interview study was conducted to explore the research question of how people with T2DM experience participating in the micro-choice-based group treatment, including its preparation phase and follow-up phase after treatment.

### The Micro-Choice-Based Concentrated Intervention

The micro-choice-based concentrated intervention for people with T2DM was part of a project collaboration between Haukeland University Hospital (Bergen, Norway) and Helse i Hardanger (Øystese, Norway). The intervention is transdiagnostic and originally developed for obsessive compulsive disorder, panic disorder, and social anxiety disorder.^18-24^ The protocol and the overall research question have been described in detail and published previously.^25^ In the present project, the concentrated format, from which the overall results have been published,^26^ was adapted to the following chronic complex disorders: low-back pain, chronic obstructive pulmonary disease, long COVID, and T2DM.

The adapted intervention for people with T2DM was delivered by an interdisciplinary team consisting of endocrinologist, psychologist, diabetes nurses, pharmacist, nutritionist, and physical therapist. The team had various specializations but overlapping expertise and collaborated closely on common goals. The intervention consisted of 3 phases, (1) a 3- to 4-week preparation phase (mostly digital), (2) a 4-day on-site group intervention, and (3) a 12-month digital follow-up. In addition to diabetes knowledge dissemination and knowledge translation, the cornerstones in the intervention were a thorough preparation of the participants to facilitate change, a focus on the many micro-choices one makes in everyday life (consciously or unconsciously), and facilitation to integrate the changes into everyday life with a minimum of follow-up from professionals.

The concept of micro-choices includes identifying when symptoms, habits, or automated and unhelpful actions occur. By targeting and intentionally breaking inappropriate patterns by making health-promoting micro-choices, the aim is to increase flexibility and a sense of being in control. In line with this, a shift in focus was emphasized from targeting glucose levels per se to targeting and monitoring the everyday micro-choices that may lead to increased insulin sensitivity (self-produced or injected). A shift away from trying to regulate the blood glucose level as such to focus on something controllable, that is, the actions (eating, activity, regulate stress, sleep, etc) that impact insulin sensitivity, has the potential to increase a sense of being self-directed in life. Furthermore, this shift implies that change is within reach and possible to control, as opposed to physiological parameters that are not under immediate or direct control, such as blood glucose levels or body weight. This shift of focus is also embedded in the attention toward food and activity during the intervention, where the emphasis is to explore how to maximize the effect of available insulin. Participants explore, among other things, how “forbidden” food and various types of physical activity affect their need of insulin and their glucose level. In this way, they can observe and evaluate the consequences of different choices. Another important feature of the intervention is goal setting. The individual goals should be specific, measurable, achievable, relevant, and time-specific (SMART goals).^27^ To achieve and maintain own goals after the concentrated intervention, the patients were guided on how to integrate the changes as part of their everyday life.^25,26^ Participants used a dedicated website for communication with health personnel and for data collection during the project.^28^

### Participants and Setting

The concentrated micro-choice-based group intervention was conducted with 11 groups from May 2021 to May 2022 for people with T2DM (N = 75). Recommended COVID-19 precautions according to the Norwegian Health Directorate were strictly followed. For the present qualitative interview study, 15 of the 75 participants were invited to participate. They all belonged to the first 4 intervention groups. All these 15 invited participants used a continuous glucose monitor (CGM) through 14 days in the preparation phase, during the on-site intervention, and through 14 days 3 months later. They were on various types of treatment regimens (insulin treatment, treatment with other glucose-lowering medications than insulin, and combinations thereof) and had various levels of diabetes-related complications and comorbidities.

Approximately 3 months after the on-site intervention, a diabetes nurse who participated in the intervention contacted the participants and gave additional information about the interview study and received verbal approval for the interviewer to contact them. The participants had previously provided written consent to participate in research studies related to the concentrated intervention, also interview studies. The interviewer (first author BEB) contacted the 15 participants to make appointments, and 14 consented to participate. Eight of the participants were female, age range was 45 to 74 years, diabetes duration ranged from 1 to 24 years, and 9 were currently employed. The participants could choose between face-to-face and telephone interview; 12 of them chose the latter. The face-to-face interviews did not include systematic participant observation. The interviews lasted from 20 minutes to 41 minutes and were all audiotaped and transcribed verbatim.

### Data Collection

A semistructured interview guide was developed by members of the research team (BEB, AH, AWL, ES). The main and follow-up topics in the interview guide are listed in Table 1. The interviews started with a broad opening question about the participants and their diabetes to familiarize them with the interview situation before the main and follow-up topics were addressed. Although the situations determined the appropriate probes to use during the interviews, some were prepared in advance. Examples of probing questions were “What do you mean?” “Talk more about that,” or “Can you describe this in more detail?” A pilot interview was conducted and later included as 1 of the 14 interviews. The pilot interview resulted in only minor adjustments in the interview guide.

**Table 1.** Topics in the Interview Guide

Aim: to describe participants’ experiences with an interdisciplinary micro-choice-based concentrated group intervention for people with type 2 diabetes.	
Main topics	Follow-up topics
Can you tell me about your diabetes?	How long have you had diabetes? How will you describe your daily life with diabetes?
Can you tell me about your participation in the concentrated intervention?	How did you experience the preparation phase? How did you experience the 4-day on-site intervention and the included tools and approaches? How did you experience participating in a group intervention? How did you experience the follow-up phase? Have you reached the goals you set for yourself? Have you experienced any changes in your life after participating? Do you have any thoughts about the future regarding the topics focused on in the concentrated intervention?

### Data Analysis

**Figure 1.**
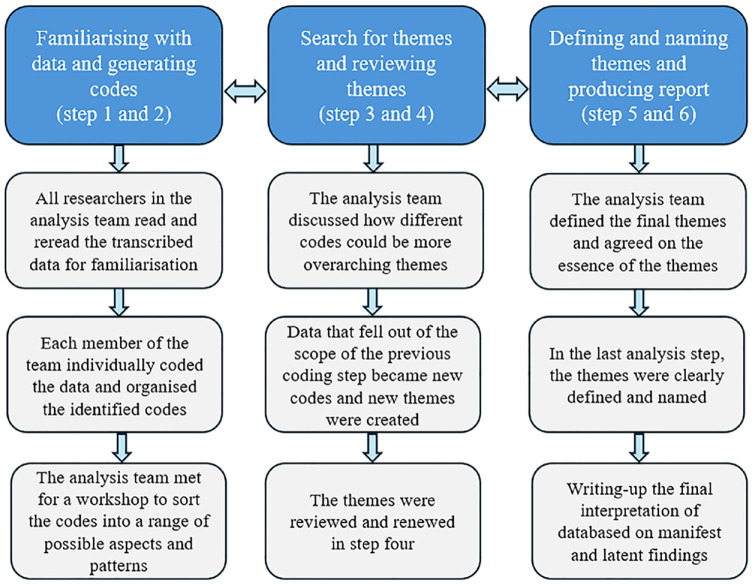
A description of the analysis process according to Braun and Clarke’s^29^ thematic analysis.

The analysis followed the 6 steps of thematic analysis described by Braun and Clarke.^29^ These are (1) familiarizing with data, (2) generating initial codes, (3) searching for themes, (4) reviewing themes, (5) defining and naming themes, and (6) producing report. Through these steps, the analysis team sought to find both manifest and latent meaning in the text.^29^ Figure 1 describes the progress of the analysis process.

In this qualitative study, trustworthiness was created by 4 criteria: credibility, dependability, confirmability, and transferability.^30^ The research team had experience and extensive knowledge of both clinical diabetes and qualitative study methodology. The researchers in the analysis team were all female, with different professional backgrounds (registered diabetes nurses: BEB, AH, EM, TLS, KL; psychologist: AWL; and endocrinologist: AS), and all had extensive diabetes knowledge. All have firsthand knowledge of providing diabetes care within primary or specialist health care services. BEB, AH, KL, and AWL have experience in diabetes research and in conducting qualitative research studies.

### Ethics

The participants were informed that participation was voluntary, that they could withdraw from the study at any time, and that only their anonymized responses would be published. The interviewer did not have any previous relationship with the participants. The data material collected was anonymized in accordance with ethical guidelines. The study was conducted in accordance with the Declaration of Helsinki and approved by the Reginal Ethics Committee for Human Research in the Western region (REK Vest 2020-101648 and 2020-203941).

## Results

The participants described their life with diabetes prior to participation in the project as a life with demanding everyday choices related to their diabetes, especially pertaining to food and activity. Some participants expressed feelings of self-reproach, shame, and guilt and that these feelings impacted their diabetes self-management negatively. Others experienced living with T2DM as manageable despite the many difficult choices.

The participants’ experiences with the interdisciplinary micro-choice-based concentrated group intervention revealed some common characteristics and patterns that are captured in the following 3 themes: (1) group intervention tailored to individual needs through friendly and skilled professionals, (2) valuable social interactions and an experience of fellowship, and (3) commitment to change through goal setting and conscious micro-choices. The themes with subthemes are shown in Table 2. In the following, accounts from each theme are demonstrated with quotes.

**Table 2.** Themes and Subthemes

Theme	Subtheme
Group intervention adapted to individual needs through friendly and skilled professionals	Readiness for change
	Important knowledge was gained through skilled professionals
	A desirable attention toward food and activity
Valuable social interactions and an experience of fellowship	Sense of community
	Exchange of experiences
	A need for individuality despite the value of group intervention
Commitment for change through goal setting and conscious micro-choices	Change of attitudes and increased awareness
	Internalized changes
	Increased engagement but still demanding

### Group Intervention Tailored to Individual Needs Through Friendly and Skilled Professionals

The participants described the participation in the concentrated micro-choice-based intervention as desirable, necessary, and positive for them. They experienced that the group intervention was tailored to their individual needs through friendly and skilled professionals, and they described a previous lack of opportunities to participate in this type of intervention. Three subthemes in this theme were identified and named: (a) readiness for change, (b) skilled professionals, and (c) a desirable attention towards food and activity.

The participants described the intervention’ preparation phase (that included a group meeting and digital follow-up with online questions) as a wake-up call about taking control of one’s own life and the need to make appropriate changes. One described:

Before the program, I kind of tried not to let it [T2DM] control me too much. I went for some walks and ate what I thought was ok, but I measured [the glucose level] very little. I didn’t measure at all because I didn’t want it to control me. I was very focused on having a normal life. (11)

During the intervention, gaining significant knowledge through skilled professionals was expressed as valuable. One said:

Yes, I think it was a very, very good treatment program. You got to learn more about both medicine and how the body works, and that you are ok even though you have diabetes. You are allowed to make micro-choices and everything . . . and that helps both psychologically and to be motivated and to feel that I can do this. (1)

Some participants realized that they lacked important diabetes knowledge or that their knowledge was old, outdated, or misleading. One said:

Because I have acquired more information than what I have found online [before the program]. Accurate and true information. Because on websites there is a lot of misinformation that scares people instead. (13)

The participants appreciated the team-based, interdisciplinary approach. Knowledge into psychology, nutrition, activity, medicine, and relevant disease control was highlighted. One said:

The competence they had, and the fact that they were available, and you could ask about everything you wanted, was extremely good. . . . I could soak up knowledge. (3)

The participants described a desirable attention toward food and activity during the intervention. The hands-on learning about food choices was experienced as valuable. In addition, they appreciated the experiments with food and physical activity in safe surroundings. These sessions increased their knowledge about how food and activity impact the body’s insulin sensitivity. Furthermore, they emphasized how the CGM had made them aware of how their glucose level was affected by food, activity, and other factors. They described how they previously had tried to restrict and regulate the meal situations but still did not reach satisfactory glucose levels. Such experiences could in the past lead to extreme actions, such as strict diets and exercise programs that they felt had a negative impact on their quality of life. One said:

I tried all kinds of diets, powder meals and living in ketosis for years, but you don’t become a nice person by doing that, . . . I don’t think I was a good mother when I was doing that. (12)

The participants described how the physical activities gave insight into how various types of activity impacted their insulin sensitivity and how through guidance, they experienced that they could participate in activities that they previously believed they could not accomplish. One participant said:

It was very positive that we had exercises every day. It was good and varied exercises. I even got really worn out when it was over. So now I exercise more than I did before. (9)

### Valuable Social Interactions and an Experience of Fellowship

The participants referred to the social interaction and experience of fellowship with peers as positive parts of the program. Three subthemes were captured and named as (a) sense of community, (b) exchange of experiences, and (c) a need for individuality.

To be together with others who could relate to participants’ own experiences created a sense of community. They described common work with something they previously experienced as a lonesome struggle. One said:

And I felt that it was nice to be together with others and we conversed well together. So, . . . we got very connected, and we experienced not being alone because we are in the same boat and have something in common that we must work with to have a good life. (11)

The intervention gave possibilities to exchange experiences, and the participants experienced that the group cheered them on. One expressed:

It was very positive to be together with others and to be able to share experiences with others. Because when you get the diagnosis . . ., I have felt that you must invent the wheel on your own. It has been nice to share experiences with each other . . . and stories. (7)

Despite the positive perception of the group format, the participants appreciated the individually tailored guidance that they received from the health care team during the intervention. The general information and medical advice they received in groups were tailored by individual advice. One participant said:

The individual focus on how I cope in daily life and how I experience living with diabetes; my own experiences and how I live my life. That was very valuable. (5)

### Commitment to Change Through Goal Setting and Conscious Micro-Choices

After the group intervention, the participants felt more committed to make changes by using goal setting and conscious micro-choices in their everyday life. The subthemes (a) change of attitudes and increased awareness, (b) internalized changes, and (c) increased engagement but still demanding, were identified.

The intervention was described as giving a boost for improved self-management through an increased awareness of several aspects of their life with diabetes. One expressed:

Yes, it [participating in the intervention] has helped me to turn my way of thinking and given me increased attention to the fact that you can’t hide this away and forget about it. I have learned to relate to the disease and take it more seriously and I kind of go all in to do the best I can now. (3)

Another said:

And now I know, because I didn’t before, now I know that if . . . by moving [activity] the insulin works, I didn’t know that before. I thought that when you injected insulin it would go down [the glucose level], but it didn’t. So now I move more. I try to walk an hour every day. (10)

Another important part of the participants’ change projects was goal setting. Their individual goals helped them to be aware of all the daily micro-choices and that further health-promoting micro-choices supported their change projects very well. To understand these relationships, CGM was experienced as an utmost valuable aid. One said:

What was really positive was learning about micro-choices. And I really would like to use a CGM [Libre] again, because it makes you understand [what impacts glucose levels] in a way you otherwise can’t. Everybody should get CGM when they get diabetes. At the time of diagnosis so you could make changes earlier. (12)

Despite positive experiences with goal setting and micro-choices, several answered that they had not made significant changes when they were asked the overarching question about whether participation in the intervention had led to specific behavior changes. But at the same time, they described valuable changes. This may be interpreted as internalized changes.

After the on-site intervention days, some participants felt like they were left on their own and felt lonesome in upholding the changes, although they had the possibility to contact the health care professionals in the program via the dedicated website. They described the everyday life as still demanding, and they expressed a wish to meet the group again. However, the participants experienced that the concentrated intervention had contributed to better quality of life, personal gain, and more consciousness about how to make healthy choices in everyday life. One expressed it this way:

Well, the most important thing I learned was that you kind of can . . . you can actually do everything, everything you want to, but that everything has consequences in a way. . . . Food was just a big problem before. And I have managed to turn that around. (3)

## Discussion

In this study, participants with T2DM described an overall positive experience with the interdisciplinary micro-choice-based concentrated group intervention. The micro-choice approach was evaluated as especially valuable. Together with increased knowledge gained through skilled professionals, it activated them and enabled them to improve their self-management through more health-promoting choices in their everyday life. Some describe the intervention as a life-changing experience.

Although not everyone felt ready to make changes in their lives with T2DM prior to enrollment, the preparation phase and an introductory meeting activated them to become prepared and committed. This essential part of the intervention was indeed meant to encourage the participants into making active choices to initiate changes.^25^ According to previous research, higher level of patient activation among people with chronic diseases is associated with favorable health-related behaviors, better quality of life, and fewer emergency department visits or hospitalizations.^11,13,31^ The concentrated intervention seemed to aid participants to an increased adherence to handling the complexity of T2DM by enhancing self-management skills and ability to translate knowledge into everyday life, specifically through micro-choices and individually developed SMART goals adapted to own preferences. Some participants described an increased activation and a change in the self-management attitudes, from feelings of shame and blame to something they actively worked with in order to take control.

The fact that the participants reported a need of more knowledge and that increased knowledge is linked to better self-management and better health outcomes is not surprising and has been shown previously.^32^ Uniquely, replacing a single-minded focus on hard numbers (ie, glucose levels, weight, cholesterol, blood pressure, etc), the concentrated intervention aimed to activate the patients to find new and better ways to adapt to a healthier life with T2DM. Findings in this study support that many people with T2DM can, according to guidelines, have a biomedically poor condition but at the same time may not experience that this has an impact in daily life because of very few symptoms and problems. This is in line with already published 3-month transdiagnostic results from the same concentrated intervention.^26^ People with T2DM had a higher baseline level of functioning than people with lower back pain or long COVID and also improved to a lesser degree after the intervention.^26^ This may indicate fewer symptoms and difficulties in daily life, which again could lead to suboptimal self-management even though the complexity and progressiveness of the condition requires change and individual activation. Participants described that they forgot to consider the management or the effect of the condition due to scant symptoms and regarded this as a reason for previously fluctuating glucose levels.

The reason for not taking the disease into account in daily life may also be linked to insufficient health literacy. Increased levels of health literacy is an important strategy so that individuals can make health-promoting choices and partake in decisions about their own health.^5^ Higher levels of health literacy are known to be associated with higher diabetes knowledge and lower levels of A1C for people with T2DM.^6^ In this light, it is highly relevant that the participants’ expressions in this study indicated that important features have been applied that may have led to increased health literacy among the participants with T2DM.

The shift from only acting on high glucose levels to making deliberate micro-choices that can increase the effect of insulin and thus avoiding high glucose levels was expressed as a new and valuable insight for the participants. This perspective might help individuals to actively make changes that will be beneficiary for metabolic control and increase active involvement. Knowledge and goal setting is frequent in behavioral change techniques for people with T2DM,^33^ but no previous studies have been found using micro-choices to enhance patient activation, other than the pilot studies performed in this intervention.^26^ In addition, all the participants in this study expressed challenges regarding eating the “right” food to avoid high glucose levels, and in this respect, the focus on micro-choices can be beneficial. The shift from attention on all the food items that one must give up toward how to facilitate the efficacy of insulin in the body can be important. This may lead to a shift from stressful attention on food toward finding tools to aid the body’s physiology.

The participants in this study described a greater understanding and better self-management after the concentrated intervention. This emphasizes the importance of meeting individual needs and tailoring information and education. In line with other studies,^14,15^ this study has shown that the group intervention was an appreciated approach for the participants, and innovative aspects into the group intervention (individualized group setting) that were regarded positively by the participants have been introduced. The concentrated format of the intervention, where the participants had 4 full days with an interdisciplinary team of health personnel and time to connect as peers, may be a new way of facilitating group-based intervention. Other studies on group training for people with T2DM typically last for a few hours over a period of several weeks or months.^15^ When the intervention is concentrated, and thus less time-consuming for participants, this can be beneficial in terms of sustaining participation during the intervention and reduced dropout. The participants in this study said that the concentrated format was positive and well arranged so that they could focus and give the intervention their full attention during the 4 days. Furthermore, the participants in this study expressed that it would be an advantage to meet the group again as a motivation to uphold changes. This could indicate a perceived need for support from peers over time, something that could be investigated in future trials.

The participants in this study claimed that the intervention’s interdisciplinary approach gave them a better understanding into specialized elements of diabetes care. A previously published systematic review has shown that people with T2DM experience satisfaction when different health care disciplines contribute to treatment and care.^34^ The concentrated format in this study was arranged so that the participants could meet the various health professionals on a one-to-one basis if needed. It was expressed by several of the participants as beneficial to be able to talk about specific aspects that were important in their life. On another note, the concentrated format of the intervention, where participants only need 3 to 4 days to participate, and the continued rehabilitation process at home may be cost-effective for participants and the health care system. The modest labor factor (10 participants; 2 group leaders) compared to traditional rehabilitation projects, often with a duration of 3 to 4 weeks or longer, may be easier to implement in daily life for participants. The concentrated micro-choice-based interdisciplinary format may thus guide practicing diabetes caregivers and educational specialists to help groups of patients with T2DM in making more health-promoting choices in their daily lives that may improve health literacy and self-management. The concentrated intervention has the potential of changing the way health care is delivered and thus could be a useful and more time-efficient addition to DSME.

### Strengths and Limitations

A preunderstanding of the studied phenomenon can be both a strength and a limitation. On the one hand, it can help researchers to collect rich and sufficient data. On the other hand, the analysis can be affected by the researchers’ own experiences of the project’s group intervention. We continuously considered the trustworthiness criteria throughout the analysis.

To limit the researchers’ preunderstanding and strengthen dependability and trustworthiness, the interviewer and 2 of the authors (KL and MMI) have not been part of the group intervention. To further strengthen the analysis collaborative coding, reflective open-minded discussions of the data were conducted during the analysis. Through a well-described design, including strategic selection of participants aiming to reach a rich variety of perceptions, confirmability was maintained. Also, use of the Standards for Reporting Qualitative Research checklist for reporting qualitative research strengthens the study’s validity.^35^

## Conclusion

This study has shown that the interdisciplinary concentrated micro-choice-based group intervention for people with T2DM was experienced as a valuable approach that met the participants’ preferences and led to self-experienced improvements in activation and diabetes self-management. The findings support the importance of enhancing individuals’ knowledge and competence in a group setting by using tools such as micro-choices and individual goal setting alongside support from an interdisciplinary team. An increased insight into the complex mechanisms involving various behaviors and the function of insulin in the body was achieved. The concept may be a key to enhanced patient activation and thereby improved self-management for people with T2DM.
